# A Nondestructive Testing Method for the Determination of the Complex Refractive Index Using Ultra Wideband Radar in Industrial Applications

**DOI:** 10.3390/s20113161

**Published:** 2020-06-02

**Authors:** Vipin Choudhary, Daniel Rönnow

**Affiliations:** 1 Department of Electrical Engineering, Mathematics and Science, University of Gävle, 801 76 Gävle, Sweden; dalrow@hig.se; 2 KTH Royal Insitute of Technology, Technical Information Science, 114 28 Stockholm, Sweden

**Keywords:** ultra wideband radar, UWB, dielectric properties, refractive index, polarimetry, wood based materials, moisture, nondestructive testing

## Abstract

An ultra-wide band radar reflection measurement technique for industrial applications is introduced. A new method for determining the complex refractive index (or equivalently the relative permittivity) of objects with planar interfaces is presented. The object thickness can also be obtained experimentally. The method is a combination of time and frequency domain techniques. The objects can be finite in size and at a finite distance. The limits in size and distance for the method to be valid are experimentally investigated. The method is relatively insensitive to hardware impairments such as frequency dependence of antennas and analog front end. The method is designed for industrial in-line measurements on objects on conveyor belts. Results are presented for solid wood and wood chips; the complex refractive index is determined in the frequency range 0.5 to 2.0 GHz for the moisture content of 3.6–10% for solid wood and 30–50% for wood chips. Polarimetric measurements are used; wood and wood chips are anisotropic.

## 1. Introduction

Ultra wideband (UWB) radar is used in several applications in non-destructive testing [1,2,3]. Free space methods are particularly suitable for industrial measurement on large volume objects. They complement techniques for determining dielectric properties commonly used in laboratory applications, in which samples of a known geometry are placed inside specifically designed cells [4] or inside waveguides [5]. In [6], a non destructive testing method using UWB signals was presented, in which a sensor detected the delay between two signals induced by a change in the dielectric property of concrete. Similarly, a method using reflected UWB signals for detecting defected and healthy carbon fiber sheets was presented in [7]. In [8,9], free space UWB methods for determining the dielectric function and thickness of multiple liquid levels in tanks are presented. In [10], the moisture content of wood chips was determined from the anisotropy of the dielectric functions using UWB radar in transmission mode. The ability of electromagnetic (EM) waves to penetrate built structures has been harnessed to determine the thickness and dielectric function of walls through UWB measurements [11]. Various building materials’ dielectric function are reported in [4,12], determined from time domain transmittance measurements. The measurement of pavement thickness and dielectric function is reported in [13]. In [14], the moisture content of grains was determined from the dielectric function in the range 3–5 GHz; a UWB sensing probe and a mode matching technique was used. Anisotropy caused by inhomogeneities on length scales shorter than the wavelength has been characterized, e.g., in [10,14].

This paper proposes a fast measurement method for determining dielectric properties of objects. Real and imaginary parts of the permittivity (or the refractive index) are determined, which makes it suitable for classification in nondestructive testing. Examples are given of solid wood (SW) and wood chips (WCs) with different moisture content and anisotropic dielectric properties. The method provides a robust in-line industrial measurement method suitable for large volumes. It can be used on objects with planar interfaces, which is common in industrial applications and makes the algorithm for determining the dielectric function simpler than for, e.g., cylindrical objects, as in [15].

The method combines time and frequency domain techniques. It is relatively numerically simple, which makes it suitable for industrial in-line applications. It complements more complex full waveform inversion techniques [16] or machine learning techniques [13] for layered media. The proposed method is insensitive to object size, and to system imperfections and measurement errors such as frequency dependence of the analog front, antenna distortions, and path loss effects. The combination of time and frequency domain makes it robust and complements frequency domain techniques [17,18]. The method for determining the imaginary part of the refractive index is to our knowledge new. It can be determined for objects of finite size and at finite distance, which makes it valuable for many practitioners. For determining the real part of the refractive index, we use the time delay of the reflected pulses, similar to, e.g., [10].

Wood-based products are tested in industrial applications, and wood can have a moisture content from 1% to 250% (depending on temperature, pressure, size, surface area, etc.) [10]. The presented method complements [10] in that it is a reflection method and consequently can be used for lower moisture levels, since the electromagnetic (EM) wave travels twice through the medium. Furthermore, both the real and imaginary parts of the refractive index (or equivalently permittivity) are determined, which enables more thorough classification in industrial applications. The presented method can be applied to anisotropic media, which is crucial for wood-based materials [10].

The method is based on the following: (1) The UWB pulses in the time domain can be separated; the pulses must be narrow enough to enable separation. (2) The objects must have materials with materials properties such that the pulses are not too distorted by scattering or multiple reflections; the frequency dependence should be moderate.

## 2. Theory

### 2.1. Wave Propagation Model

**Figure 1:**
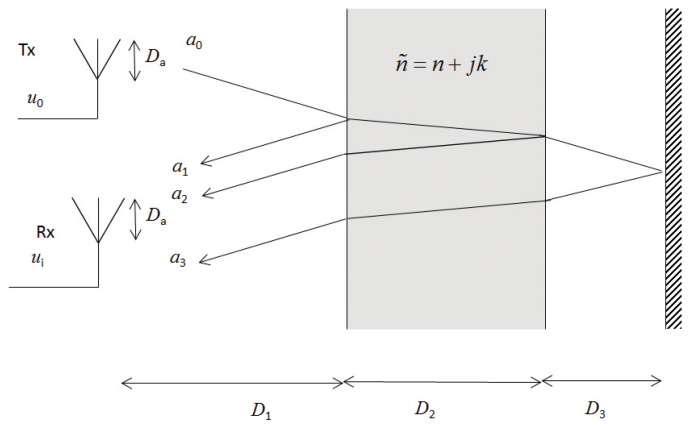
The geometry of the experiment for determining the time delay of the reflected pulse. Tx and Rx are the transmit and receive antenna. $D_{1}$, $D_{2}$, and $D_{3}$ are the distances between the antennas and the reflecting interfaces; $a_{0}$ is the pulse emitted from the Tx antenna, and $a_{1}$, $a_{2}$, and $a_{3}$ are the pulses from the first reflections in the respective interfaces and reach the receive antenna. $u_{0}$ is the reference pulse, and $u_{i}$ (i = 1, 2, 3, …, r) represents the recorded pulses. The object’s refractive index is $\tilde{n}=n+jk$. At $D_{3}$, there is a reference object.

**Figure 2:**
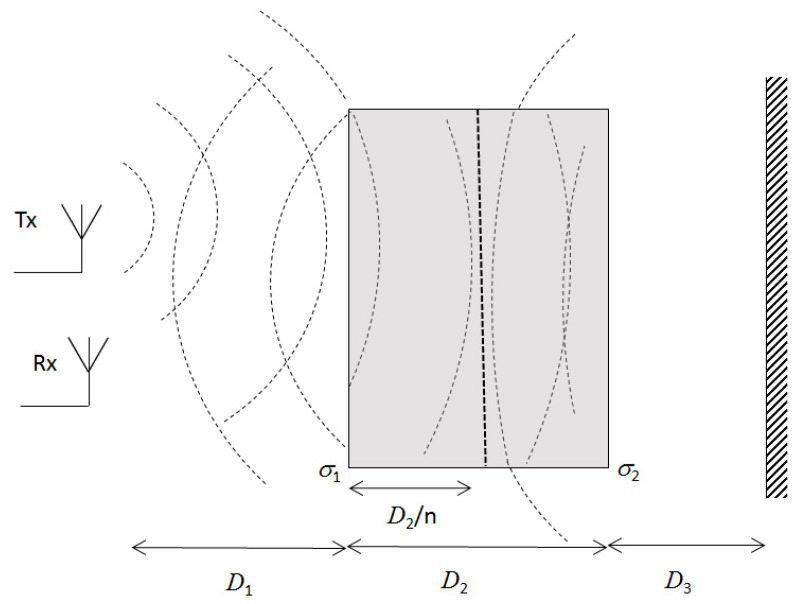
Illustration of the different path loss effects that affect the amplitude of the measured pulses.

We assume homogeneous linear media without any ferro- or ferri-magnetic properties. The assumption of homogeneous media means that any heterogeneities must be on length scales much smaller than the wavelength of the electromagnetic radiation, i.e., an effective medium [19]. The electromagnetic interaction is then described by the complex relative dielectric permittivity $\tilde{\varepsilon }=\varepsilon^{\prime }-j\varepsilon^{\prime \prime }$ (or the complex refractive index $\tilde{n}=n+jk$, where $\varepsilon^{\prime }=n^{2}-k^{2}$ and $\varepsilon^{\prime \prime }=2nk$). We assume that the objects have smooth interfaces, such that they reflect the radiation specularly. This assumption is valid if the objects are large compared to the wavelength and the roughness is much smaller than the wavelength [20]; this is valid for many industrial surfaces. The reflections in the planar interfaces are described by the Fresnel’s formulae [21]. The geometry of the experiment is shown in Figure 1 and Figure 2, where Figure 1 illustrates the reflection in the different interfaces, and Figure 2 shows the wave propagation and how the different interfaces as a consequence of refraction are illuminated differently. In an alternative geometry, the object is placed directly at the reference, in which case $D_{3}=0$. The radar pulse, $a_{0}$, is emitted by the Tx antenna; reflected pulses, $a_{1}$, $a_{2}$, and $a_{3}$, from the different interfaces are received by the Rx antenna. The distance between the antennas and the object is $D_{1}$, the object’s thickness is $D_{2}$, and the distance from the object to the reflector is $D_{3}$.

The distance from the antennas is large enough such that the objects are not in the reactive near field region, but in the Fresnel region or beyond, where the E- and H-fields are perpendicular, i.e., $D_{1}>2D_{a}^{2}/\lambda$, where $D_{a}$ is the antennas’ maximum dimension, and λ is the wavelength of the electromagnetic radiation [22]. $D_{2}/n$ is the apparent distance from Interface 1 to Interface 2, as given by Snell’s law for refraction and the small angle approximation. The frequency dependence of the complex refractive index, $\tilde{n}=n+jk$, is assumed to be small, such that an effective frequency independent refractive index can be used within the experimental bandwidth; such an approximation is motivated by the small frequency dependence of several materials used in industrial applications, such as wood [23], plastic [24,25], concrete [26], and glass [27]. For liquid water, the dielectric function has a resonance around 20 GHz [28]. At lower frequency—where the absorption is smaller—, the frequency dependence is moderate. The refractive index of ice is practically flat in the region 0.1 to 300 GHz [29]. Frequency dependence in *n* and *k* are related through Kramers-Kronig relations [30]. Furthermore, we investigate materials for which n>>k, in which case the EM-wave will propagate through the object and pulses due to reflections in the different interfaces will be detectable at the Rx antenna. The reflection of EM-waves by stratified media is well studied in, e.g., optics [21], in which case an infinite number of multiple reflections are combined. We use the same approach, but we do not consider multiple reflections, since in our case we can separate the different reflected pulse in the time domain. This approach has been used for UWB radar signals in stratified media [8,11,31].

The used Vivaldi antennas have wide lobes and the radiated field is close to isotropic [10]. The reflecting interfaces of the object are not always at the same distance, and the reflecting area may vary between objects. Hence, the power of the radiation reaching the object varies. Furthermore, parts of the reflected radiation from the second interface will not be transmitted through the first interface, but through the sides of the object (see Figure 2). In the case of the object’s interface going to infinity in both directions, all radiation would be mirrored. A path loss model with the amplitude $\propto D_{1}^{-1}$ would be used as in [31]. If the illuminated were finite but the distance goes to infinity, a path loss model with the amplitude $\propto D_{1}^{-2}$ would be used as in the “radar equation,” see, e.g., Chapter 14 in [32]. In our case, the objects have finite areas and are at finite distance and we use a path loss model where the amplitude is $\propto D_{1}^{-\gamma }$, where 1≤γ≤2 and γ is different for different objects and distances.

The measured, transmitted, and received pulses are related by

(1) $$a_{0}\left(t\right)=u_{0}\left(t\right)*g_{t}\left(t\right),$$

(2) $$u_{i}\left(t\right)=a_{i}\left(t\right)*g_{r}\left(t\right),$$

where $g_{t}\left(t\right)$ is the Tx antenna’s impulse response, $g_{r}\left(t\right)$ is the Rx antenna’s impulse response, $u_{0}$ and $u_{i}$ are the emitted and detected EM-pulses, and ∗ denotes the convolution. $g_{t}$ and $g_{r}$ also include the effects of the analog front end. For the pulse reflected in the first interface and received at the Rx antenna we obtain

(3) $$a_{1}\left(t\right)=\frac{a_{0}\left(t-\tau_{1}\right)}{{\left(2D\right)}^{\gamma_{1}}}*\sqrt{\sigma_{1}},$$

(4) $$\tau_{1}=\frac{2D_{1}}{c_{0}},$$

where $\sigma_{1}$ is the radar cross section (RCS) for Interface 1 of the object, $\tau_{1}$ is the time delay of $a_{1}$, and $c_{0}$ is the speed of light in vacuum, and $\gamma_{1}$ is the path loss coefficient for the wave propagating between the antennas and Interface 1. For the pulse reflected in the second interface, we obtain

(5) $$a_{2}\left(t\right)=\frac{a_{0}\left(t-\tau_{2}\right)*t_{12}*t_{21}*\alpha_{12}*\alpha_{21}}{{\left(2\left(D_{1}+\frac{D_{2}}{n}\right)\right)}^{\gamma_{2}}}*\sqrt{\sigma_{2}},$$

(6) $$\tau_{2}=\frac{2(D_{1}+nD_{2})}{c_{0}},$$

where $t_{12}$ and $t_{21}$ are the Fresnel transmission coefficients, and $\sigma_{2}$ is the radar cross section of Interface 2 of the object, and $\alpha_{12}$ and $\alpha_{21}$ is the attenuation of the wave propagating through the object between Interface 1 and 2. $\tau_{2}$ is the time delay of pulse $a_{2}$, and the term $nD_{2}$ is due to the change of the phase velocity of the EM-wave in the medium. The term $D_{2}/n$ describes that the apparent distance from Interface 1 and Interface 2 is changed by the refraction in Interface 1, and this affects the received signal’s amplitude at the Tx antenna. In the same way, we obtain for $a_{3}$,

(7) $$a_{3}\left(t\right)=\frac{a_{0}\left(t-\tau_{2}\right)*t_{12}*t_{23}*t_{32}*t_{21}*\alpha_{12}*\alpha_{21}}{{\left(2\left(D_{1}+\frac{D_{2}}{n}+D_{3}\right)\right)}^{\gamma_{3}}}*\sqrt{\sigma_{r}},$$

(8) $$\tau_{3}=\frac{2(D_{1}+nD_{2}+D_{3})}{c_{0}}.$$

In a reference measurement, the object is removed and the received waveform is

(9) $$a_{r}\left(t\right)=\frac{a_{0}\left(t-\tau_{r}\right)}{{\left(2\left(D_{1}+D_{2}+D_{3}\right)\right)}^{\gamma_{r}}}*\sqrt{\sigma_{r}},$$

(10) $$\tau_{r}=\frac{\left(D_{1}+D_{2}+D_{3}\right)}{c_{0}},$$

where $\sigma_{r}$ is the radar cross section of the reference target.

### 2.2. Anisotropy and Polarimetry

An anisotropic material exhibits different dielectric properties for different directions of an exiting EM-field. We use the Jones matrix formalism to analyze EM-waves’ interaction with such materials [21], which is formulated in the frequency domain. The transmitted signal is described as a vector with the components related to the used coordinate system. The pulses from the Tx antenna are

(11) $$A_{0}=\begin{array}{c} \left[\begin{array}{c} A_{0,\|}\left(f\right)\\ A_{0,⊥}\left(f\right) \end{array}\right] \end{array},$$

where $A_{0,⊥}\left(f\right)$ and $A_{0,\|}\left(f\right)$ are the Fourier transforms of the perpendicular and parallel components of $a_{0}$, respectively. The propagation through a medium and the reflection or transmission in an interface are described by matrices. The pulse $a_{1}$ is described by

(12) $$A_{1}=P_{1}R_{12}P_{1}A_{0}$$

where

(13) $$P_{1}=\begin{array}{c} \left[\begin{array}{cc} e^{-j2\pi f(\frac{\tau_{1}}{2})} & 0\\ 0 & e^{-j2\pi f(\frac{\tau_{1}}{2})} \end{array}\right] \end{array}$$

describes the propagation through air between the antennas and the first interface, and

(14) $$R=\begin{array}{c} \left[\begin{array}{cc} r_{12,\|\|} & 0\\ 0 & r_{12,⊥⊥} \end{array}\right] \end{array}$$

describes the reflection of the EM-wave in Interface 1 by the Fresnel coefficients $r_{12,\|\|}$ and $r_{12,⊥⊥}$. Furthermore, matrices $A_{2}$ and $A_{3}$ can be described in a similar way.

The signals $u_{0}\left(t\right)$ and $u_{r}\left(t\right)$ are scalar. Their Fourier transforms are related to the Jones vectors $A_{0}$ and $A_{r}$. For $A_{0}$ and $U_{0}$, we write

(15) $$A_{0}=G_{t}U_{0}=\begin{array}{c} \left[\begin{array}{c} G_{t,\|}\\ G_{t,⊥} \end{array}\right] \end{array}U_{0},$$

where $G_{t,\|}=G_{t}cos\left(\theta -\theta_{0}\right)+G_{t,c}$, and $G_{t,⊥}=G_{t}sin\left(\theta -\theta_{0}\right)+G_{t,c}$; $G_{t}$ is the antenna gain, θ is the rotation angle of the transmit antenna, $\theta_{0}$ is an offset in the rotation angle, and $G_{t,c}$ is the cross-talk that describes that the antenna may transmit an EM-wave perpendicular to its plane. In the same way for the received signal $U_{i}$, we write

(16) $$U_{i}=\begin{array}{c} \left[\begin{array}{cc} G_{r,\|} & G_{r,⊥} \end{array}\right] \end{array}A_{i},$$

where $G_{r,\|}=G_{r}cos\left(\theta -\theta_{0}\right)+G_{r,c}$, and $G_{r,⊥}=G_{r}sin\left(\theta -\theta_{0}\right)+G_{r,c}$; $G_{r,c}$ represent the corresponding angles and crosstalk for the receive antenna. Notice that $\theta_{0}\approx 0$, $G_{t,c}<<G_{t}$, and $G_{r,c}<<G_{r}$, and we assume that $G_{r}=G_{t}$, since we use the same type of Vivaldi antenna for both the Tx and Rx antennas.

### 2.3. Refractive Index Determination

In the presented method, the pulses reflected from the different interfaces of the object must be separable in time. We do not consider multiple reflections; such pulses are small in amplitude and seen at longer times than the three initial pulses. For the method to work, the reflected pulses from Interfaces 1 and 2 must be separable in time, i.e., $\tau_{2}-\tau_{1}>\tau_{sep}$, where $\tau_{sep}$ is the smallest time between two pulses that enable separation. Typically, $\tau_{sep}$ is the same as or slightly larger than the pulse width:

(17) $$\frac{nD_{2}}{c_{0}}\leq \tau_{sep}\approx \tau_{pulse}.$$

**Figure 3:**
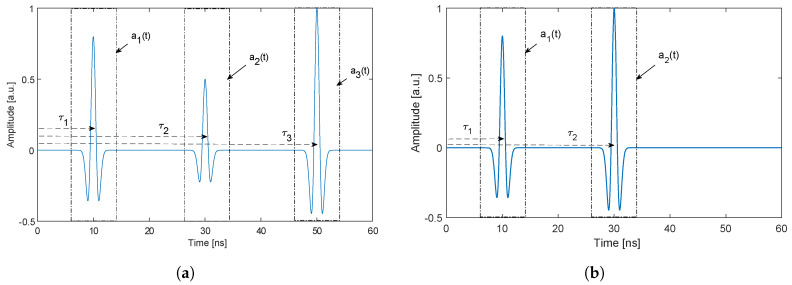
Schematic illustration of a detected time domain signal, with the three reflected waves, *a*_1_, *a*_2_, and *a*_3_, at times *τ*_1_, *τ*_2_, and *τ*_3_. The part illustrates the time windows and stating times used in the analysis. A signal (**a**) with three interfaces, and (**b**) with two interfaces (*D*_3_ = 0), where the reference object is at the second interface.

Figure 3 illustrates the detected time domain pulses used to determine the refractive index. Pulses are detected in the recorded time series in the following steps:

1. Find the maximum value and the corresponding time instants of the time series. Define a time interval around this time instant; this is now one pulse.
2. Find the maximum value and the corresponding time instant of the time series, without the time interval from Step 1.
3. Continue until two ($D_{3}=0$) or three ($D_{3}\neq 0$) pulses have been detected.
4. Sort the pulses in adjacent order in time such that pulse $a_{1}$, $a_{2}$, and $a_{3}$ are obtained.

The real part of the refractive index, *n*, is determined from time delays. The time delay of the reflected pulses is determined by the first zero-crossing before the main peak, which is an established method for robust identification of time delays of UWB radar pulses [1]. Combining Equations (4), (6), (8) and (10), we obtain

(18) $$D_{1}=\frac{c_{0}\tau_{1}}{2},$$

(19) $$D_{2}=\frac{c_{0}\left(\tau_{r}-\tau_{1}+\tau_{2}-\tau_{3}\right)}{2},$$

(20) $$D_{3}=\frac{c_{0}\left(\tau_{3}-\tau_{2}\right)}{2},$$

(21) $$n=\frac{\tau_{2}-\tau_{1}}{\tau_{r}-\tau_{1}+\tau_{2}-\tau_{3}}.$$

To determine the imaginary part, *k*, of the refractive index, we use the Fourier transform of each detected pulse. We obtain from Equation (2)

(22) $$U_{i}\left(f\right)=A_{i}\left(f\right)G_{r}\left(f\right),$$

(23) $$U_{r}\left(f\right)=A_{r}\left(f\right)G_{r}\left(f\right),$$

where $A_{r}\left(f\right)$ is the Fourier transform of $a_{r}\left(t\right)$, etc., and we get

(24) $$\frac{A_{i}\left(f\right)}{A_{r}\left(f\right)}=\frac{U_{i}\left(f\right)}{U_{r}\left(f\right)}.$$

Further, with the help of Equations (3)–(10), the received pulses can be analyzed step by step for each interface (cf. Figure 1) in the frequency domain; the received signal contains reflected and transmitted coefficient of the EM-wave’s response to different interfaces. Initially, we analyze the ratio of the Fourier transforms of $u_{1}$ and $u_{r}$, and using Equations (3), (9) and (24), we obtain

(25) $$\frac{A_{1}\left(f\right)}{A_{r}\left(f\right)}=\frac{{\left(2\left(D_{1}+D_{2}+D_{3}\right)\right)}^{\gamma_{r}}}{{\left(2\left(D_{1}\right)\right)}^{\gamma_{1}}}\frac{R_{12}\Omega_{1}}{R_{r}\Omega_{r}}e^{\left(-j2\pi f\left(\tau_{1}-\tau_{r}\right)\right)},$$

where $\sqrt{\sigma_{1}}=R_{12}\Omega_{1}$ and $\sqrt{\sigma_{r}}=R_{r}\Omega_{r}$ with $R_{12}$ and $R_{r}$ being the Fresnel reflection coefficients for Interface 1 and the reference, respectively, $\Omega_{1}$ and $\Omega_{r}$ model the illuminated area of the respective interface and the losses due to, e.g., shadowing.

For $u_{2}$ and $u_{r}$, we obtain the corresponding ratio:

(26) $$\frac{A_{2}\left(f\right)}{A_{r}\left(f\right)}=\frac{{\left(2\left(D_{1}+D_{2}+D_{3}\right)\right)}^{\gamma_{r}}}{{\left(2\left(D_{1}+\frac{D_{2}}{n}\right)\right)}^{\gamma_{2}}}\frac{T_{12}R_{23}T_{21}\Omega_{2}}{R_{r}\Omega_{r}}e^{\left(-j2\pi f\left(\tau_{2}-\tau_{r}\right)\right)}e^{\left(-4\pi fD_{2}\frac{k}{c_{0}}\right)},$$

where $T_{12}$ and $T_{21}$ are the Fresnel transmission coefficients, and we have used the Fourier transform of absorption coefficients FT$\left(\alpha_{12}\right)$ = FT$\left(\alpha_{21}\right)$=$e^{\left(-\omega D_{2}\frac{k}{c_{0}}\right)}$ (cf. Chapter 4 in [21]). Similarly, the ratio of $u_{3}$ to $u_{r}$ and $u_{3}$ to $u_{1}$ becomes

(27) $$\frac{A_{3}\left(f\right)}{A_{r}\left(f\right)}=\frac{{\left(2\left(D_{1}+D_{2}+D_{3}\right)\right)}^{\gamma_{r}}}{{\left(2\left(D_{1}+\frac{D_{2}}{n}+D_{3}\right)\right)}^{\gamma_{3}}}\frac{T_{12}T_{23}R_{r}T_{32}T_{21}\Omega_{3}}{R_{r}\Omega_{r}}e^{\left(-j2\pi f\left(\tau_{3}-\tau_{r}\right)\right)}e^{\left(-4\pi fD_{2}\frac{k}{c_{0}}\right)},$$

and

(28) $$\frac{A_{3}\left(f\right)}{A_{1}\left(f\right)}=\frac{{\left(2\left(D_{1}\right)\right)}^{\gamma_{1}}}{{\left(2\left(D_{1}+\frac{D_{2}}{n}+D_{3}\right)\right)}^{\gamma_{3}}}\frac{T_{12}T_{23}R_{r}T_{32}T_{21}\Omega_{3}}{R_{12}\Omega_{1}}e^{\left(-j2\pi f\left(\tau_{3}-\tau_{1}\right)\right)}e^{\left(-4\pi fD_{2}\frac{k}{c_{0}}\right)}.$$

Notice that in Equation (27) for the case that $D_{3}=0$, $R_{23}=R_{r}$, $T_{32}=T_{23}=1$, $\tau_{3}=\tau_{2}$, it becomes the same as Equation (26). In Equations (26)–(28), $|R_{12}|=|R_{23}|=\frac{|1-n|}{|1+n|}$, $|T_{12}|=\frac{2}{|1+n|}$, and $|T_{21}|=\frac{2n}{|1+n|}$, and the frequency dependence is assumed to be negligible.

We take the logarithm of Equation (27) and obtain

(29) $$\begin{array}{c} \underset{Y_{3r}}{\underset{︸}{ln(\frac{|A_{3}\left(f\right)|}{|A_{r}\left(f\right)|})}}=\underset{C_{3r}}{\underset{︸}{\gamma_{r}ln\left(2\left(D_{1}+D_{2}+D_{3}\right)\right)-\gamma_{2}ln\left(2\left(D_{1}+\frac{D_{2}}{n}+D_{3}\right)\right)+\frac{ln(T_{12}T_{23}R_{r}T_{32}T_{21}\Omega_{3})}{ln(R_{r}\Omega_{r})}}}\\ -\underset{B_{3r}}{\underset{︸}{\frac{4\pi D_{2}k}{c_{0}}}}f. \end{array}$$

The imaginary part of the refractive index, *k*, is obtained from the slope

(30) $$k=\frac{B_{3r}c_{0}}{4\pi D_{2}}.$$

In the same way, we derive

(31) $$\begin{array}{c} \underset{Y_{2r}}{\underset{︸}{ln(\frac{|A_{2}\left(f\right)|}{|A_{r}\left(f\right)|})}}=\underset{C_{2r}}{\underset{︸}{\gamma_{r}ln\left(2\left(D_{1}+D_{2}+D_{3}\right)\right)-\gamma_{2}ln\left(2\left(D_{1}+\frac{D_{2}}{n}\right)\right)+\frac{ln(T_{12}R_{23}T_{21}\Omega_{2})}{ln(R_{r}\Omega_{r})}}}-\underset{B_{2r}}{\underset{︸}{\frac{4\pi D_{2}k}{c_{0}}}}f, \end{array}$$

(32) $$\underset{Y_{1r}}{\underset{︸}{ln(\frac{|A_{1}\left(f\right)|}{|A_{r}\left(f\right)|})}}=\underset{C_{1r}}{\underset{︸}{\gamma_{r}ln\left(2\left(D_{1}+D_{2}+D_{3}\right)\right)-\gamma_{1}ln\left(2D_{1}\right)+ln\left(R_{12}\Omega_{1}\right)-ln\left(R_{r}\Omega_{r}\right)}},$$

and

(33) $$\underset{Y_{31}}{\underset{︸}{ln(\frac{|A_{3}\left(f\right)|}{|A_{1}\left(f\right)|})}}=\underset{C_{31}}{\underset{︸}{\gamma_{1}ln\left(2D_{1}\right)-\gamma_{2}ln\left(2\left(D_{1}+\frac{D_{2}}{n}+D_{3}\right)\right)+\frac{ln(T_{12}T_{23}R_{r}T_{32}T_{21}\Omega_{3})}{ln(R_{1}\Omega_{1})}}}-\underset{B_{31}}{\underset{︸}{\frac{4\pi D_{2}k}{c_{0}}}}f,$$

where, in Equations (29), (31) and (33), $B_{3r},B_{2r}$, and $B_{31}$ are non-zero, whereas, in Equation (32), $B_{1r}=0$, which means that the slope is zero. Further, if $D_{3}=0$, Equation (29) can be rewritten as Equation (33), which is same as the case when pulses $a_{1}$ and $a_{2}$ are analyzed with 2 interfaces. Notice that the refractive index is determined for each polarization state (cf. Section 2.2); i.e., there is a $Y_{3r}$ for the ‖‖ case and one for the ⊥⊥ case.

### 2.4. Error Analysis

We assume that the errors, $\delta_{\tau }$, in the determined time delays, $\tau_{1},\tau_{2},\cdots \tau_{r}$, are independent and thus added, that $\delta_{\tau_{i}}<<\tau_{i}$, and that $\delta_{\tau_{i}}$ is the same for all pulses $a_{i}$. The error is caused by transmitter and receiver noise and clock jitter. A conventional error analysis (see, e.g., [33]) of Equation (19) gives the relative error in the determined thickness:

(34) $$\frac{\delta D_{2}}{D_{2}}=\frac{4\delta_{\tau }}{\left(\tau_{r}-\tau_{1}+\tau_{2}-\tau_{3}\right)}.$$

In the same way, the relative error in the refractive index, *n*, in Equation (21) becomes

(35) $$\frac{\delta n}{n}=\frac{2\delta_{\tau }}{\left(\tau_{2}-\tau_{1}\right)}.$$

The error, $\delta B_{2r}$, in the coefficient $B_{2r}$, is obtained from the least squares fit of the straight line (see, e.g., [33]). Using that, in Equation (36), we obtain

(36) $$\frac{\delta k}{k}=\frac{\delta B_{2r}}{B_{2r}}+\frac{\delta D_{2}}{D_{2}}.$$

Notice that, since a ratio is taken in Equation (31), systematic errors that are linear in amplitude will cancel; noise is included in the error in Equation (36).

## 3. Experiment

The measurements were carried out using a radar system in a controlled laboratory environment.

### 3.1. Radar System and Setup

**Figure 4:**
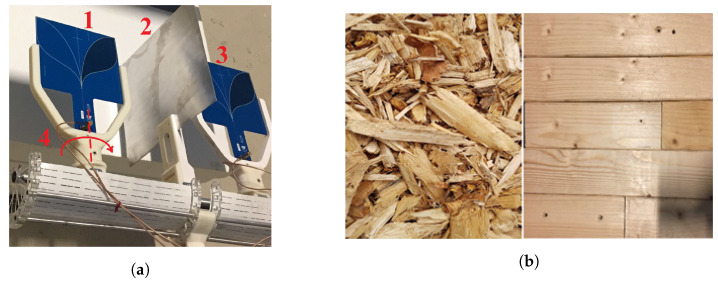
(**a**) Radar unit: (1) Tx Vivaldi antenna, (2) shielding plate, (3) Rx Vivaldi antenna, and (4) rotatory axis. (**b**) Samples: Wood chips (WCs) (left) and solid wood (SW) (right).

A UWB radar unit [34] equipped with linearly polarized antennas that can be rotated was used to perform measurements in different polarization modes (see Figure 4a). It operates in the frequency range of 0.5–2 GHz (corresponding to the wavelength range 0.5–0.15 m). The antennas are linearly polarized Vivaldi antennas that can be manually rotated for different polarization modes (such as ⊥⊥, ⊥‖, ‖⊥, and ‖‖). In the laboratory environment, the measurement setup was arranged to correspond to two different industrial test cases:

- The objects were placed horizontally and were illuminated horizontally with a flat reference reflector behind the object. The objects were put 1 m above the ground on Styrofoam blocks (with ε≈1) to minimize reflections from the ground surface. This case corresponds to an industrial application, which requires an investigation of dielectric properties of materials contained in boxes or cases with a conveyor belt with, e.g., piles of wood being tested in-line horizontally (Figure 4b). The distance between the object and the reference is arbitrary, but $D_{3}\neq 0$.
- The objects were on a conveyor belt with the radar unit illuminating the samples vertically from above. This case corresponds to an industrial application in which, e.g., wood chips are tested in-line (Figure 4b). In this case, the object is adjacent to the reference, and $D_{3}=0$.

### 3.2. Objects

For the study, we used piles of SW planks and debarked WCs, and the latter were of the type used in district heating [10]. These SW and WC samples were mainly from hard pine wood and fragile bark WCs, respectively, which are common types of wood used in the manufacturing industries and district heating in Sweden. The experiments were carried out on different types of objects:

- Piles of planks of SW inside the test boxes were used (used in Test Setup I, in Section 3.1):
  Hard pine wood was cut into small pieces of piles of SW planks to fit in plastic boxes. The dimensions of SW planks were 50–60 mm in width, 20–35 mm in thickness, and 100–400 mm in length. The SW was placed inside the plastic boxes in such a way that the orientation of wood fibers were in one direction, i.e., parallel to the ground, with no spacing in between the SW planks, as shown in Figure 4b. The dimensions of the plastic boxes were 60 cm × 30 cm × 30 cm, and the thickness of the box’s wall was very small (2 mm) compared to the operating wavelength.
- WCs inside test boxes were investigated (used in Test Setup I, in Section 3.1):
  The WCs were filtered to remove dust or sand before filled into the plastic boxes. The dimensions of the chips were 1.5–20 mm in width/thickness and 22–70 mm in length. The chips were manually distributed inside the box, which gives a random orientation of the WCs in the horizontal plane. Gravity oriented the chips such that an anisotropic medium was obtained [10]. The dimensions and the wall thickness of plastic boxes were the same as above. Note that no external pressure was applied to the WCs inside the box during the filling process.
- Piles of SW planks on the conveyor belt were investigated (used in Test Setup II, in Section 3.1):
  Piles of planks of SW were placed directly on the conveyor belt. The dimensions of SW were as mentioned above. The arrangement of SW on the conveyor belt was such that the orientation of wood fibers are in one direction, i.e., parallel to the conveyor-belt’s length. In this case, there is no specific geometry of volume of piles of SW planks, but the height is in the range of 30–40 cm. A standard industrial size of the conveyor belt with a width of 2 m, a thickness of 30 mm, and a length limited to 2 m was used.
- WCs on the conveyor belt were used as the last type of object (used in Test Setup II, in Section 3.1):
  Bark WCs were placed directly on the conveyor belt. The dimensions of the WC were the same as above. WCs were manually distributed on the conveyor-belt in such a way that the orientation of the WCs was random in the horizontal plane, but oriented by gravity to yield an isotropic medium [10]. In this case, there was no specific geometry of the volume of WC samples, but the height was in the range of 20–40 cm. A standard industrial size of conveyor belt with aforementioned dimensions was used.

The SW and WCs used for different types of objects were at room temperature during the experiments. However, SW and WCs have an uneven moisture content depending on treatment, environment humidity, and storage conditions. WCs absorb more water than SW when exposed to moist or wet conditions. Bark WCs can contain a moisture content in a range from 20% to 200%, because of its complex structure with chips with a large surface-to-volume ratio and fibers of different orientation. The dielectric properties of SW and WCs are directly dependent on the moisture percent, which may require complex analysis of the refractive index, in particular, absorption (i.e., *k*). Therefore, the presented model can be used to determine a complex refractive index of mentioned dissimilar objects with high accuracy.

### 3.3. System Characterization

Initially, a number of measurements were performed to characterize the radar system, which includes the following:

1. Variation of the distance to the objects, with objects of different size. The objects were metallic sheets of size varying from 10 × 10 cm${}^{2}$ to 200 × 200 cm${}^{2}$. Te distance was varied from 20 to 150 cm.
2. Repeatability was tested by 20 consecutive measurements with a metallic sheet of 100–100 cm${}^{2}$ at a distance of 30 cm.

**Figure 5:**
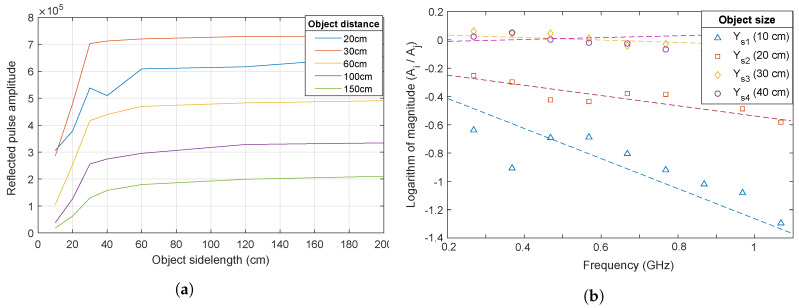
Experimental values of (**a**) amplitude vs. object side length for different object distance, and (**b**) $Y_{s1}=ln(|\frac{A_{10}}{A_{r}}|),Y_{s2}=ln(|\frac{A_{20}}{A_{r}}|),Y_{s3}=ln(|\frac{A_{30}}{A_{r}}|),\;\mathrm{and}\;Y_{s4}=ln(|\frac{A_{40}}{A_{r}}|)$ vs. frequency, where *A*_10_, *A*_20_, *A*_30_, and *A*_40_ represent pulses from objects with 10 cm, 20 cm, 30 cm, and 40 cm side lengths respectively, and *A_r_* is the reference pulse (dashed lines represent least square fits).

In Figure 5a, the peak amplitude of the reflected pulse amplitude vs. the object side length is shown for different object distances. Initially, the amplitude increases with the object side length, as expected, since the RCA increases with object size. At longer side lengths, the amplitude becomes constant; the objects are so large that practically all transmitted radiation is reflected by the object. In the same figure, it is seen that the amplitude changes with respect to distance, i.e., initially the amplitude increases with increases in distance at the same object side length (see the blue line with a 20 cm and the red line with a 30 cm object distance), and it then begins to decrease (with $1/D^{2},D$ is distance, 30 cm, from the radar) due to near-field and far-field regions of antenna radiation, which is discussed in depth in [22].

Figure 5b shows the logarithm of the ratio of the amplitude of the reflected pulse to the reference pulse vs. the frequency. It can be clearly seen in the figure that, for the object with side length of 10 cm, the ratio is strongly frequency-dependent and, for the object a 20 cm side length, the ratio shows some frequency dependence. For the objects with 30×30 cm${}^{2}$ and 40×40 cm${}^{2}$ sizes, the ratio is close to constant, which means that the ratio is frequency-independent. The negligible frequency dependence for objects with side lengths of 30 cm and larger shows that this object size is needed for the derivations in Section 2.3 to be valid. Notice that we assume that the system’s frequency dependence and imperfections (such as antenna frequency dependence and filtering of the signal through wires) are the same throughout all measurements. Therefore, these imperfections were cancel out under the logarithm of the ratio of the amplitude of the reflected pulse to the reference pulse in the frequency domain.

The error, $\delta_{\tau }$, was estimated from the measurement repeatability. The measurements were carried out within short time intervals on objects that were 100×100 cm${}^{2}$ metallic sheets, which were placed at a distance of 30 cm from the radar. The time delay of each signal was determined, and the standard deviation was determined. The error $\delta_{\tau }$ obtained is $0.03\times 10^{-9}$ s, which is mainly due to changes in clock jitters, thermal noise, and temperature variation of the analog front end.

### 3.4. Experiment Preparation and Procedure

**Figure 6:**
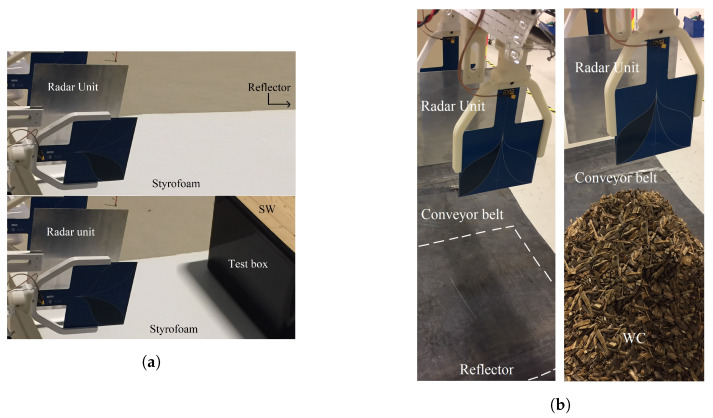
Photographs of (**a**) Setup I: reference measurement (top) and measurement of Object A (bottom). (**b**) Setup II: reference measurement (left) and measurement of Object D (right).

Figure 6 shows setup I and setup II for reference measurements and object measurements. We know that the samples have uneven moisture content even at room temperature, as discussed in Section 3.2 (see above). The samples were initially dried in a conventional microwave oven for 1 to 8 h, to achieve different moisture levels. The samples were subsequently placed in the open-lid plastic container and mixed manually during the drying process to ensure that the samples were evenly dried and after that the lid was closed to avoid ambient moisture. The moisture content of SW and WCs was determined using standard industrial process as follows: (a) take 7–8 random samples of two kilograms each, (b) mix all samples well in a plastic container and pick a random sample of 2 kg, (c) weigh the selected moist sample, (d) dry it, (e) re-weigh the dry sample after the drying process, and (f) calculate the moisture percent.

Initially, reference measurements $a_{r}\left(t\right)$ were made on a reference object for each antenna orientation, before the measurements on the objects were made. Furthermore, the objects were then positioned one by one in between the radar housing and the metallic sheet as a reflector. The measurements, $u_{i}\left(t\right)$, were performed using Setup I for all different antenna orientations and different moisture content values of SW and WCs. In Setup II, a reference measurement $a_{r}\left(t\right)$ was made, and the position of a reference reflector (i.e., a metal sheet reflector) was placed below the conveyor belt with $D_{3}=0$. The samples were manually distributed on the conveyor-belt and mixed well, in order to achieve uniform moisture distribution.

## 4. Results

**Figure 7:**
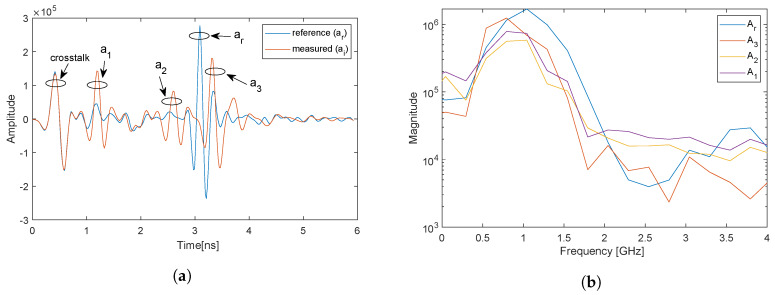
(**a**) Measured time domain signals. (**b**) Magnitude of frequency domain signals.

Examples of measured signals and a reference signal are shown in Figure 7a. The magnitude of the Fourier transform of the different pulses after using the time windowing technique (see Section 2.3) are shown in Figure 7b.

For the reference measurement in Figure 7a, we see two clear pulses; the first is due to antenna cross talk and the second is the reflected pulse from the reference object. For the measurement with an object, we see three clear pulses that are from different interfaces, in addition to the antenna cross talk. In Figure 7b, the magnitude spectra of the pulses show that the usable bandwidth is 0.5–2 GHz.

### 4.1. Boxes

Measurements were performed on SW (Object A above) and WCs (Object B above, horizontally illuminated (see Setup I above), used to determine the complex refractive index (i.e., the real and imaginary parts). In this case, $D_{3}\neq 0$, and the real part, *n*, and the imaginary part, *k*, were determined for ⊥⊥ and ‖‖ polarization states, where *n* was calculated from Equation (21) and *k* from the slope of $Y_{3r}$, $Y_{1r}$, and $Y_{31}$, as described in Section 2.2 and Section 2.3. Notice that this case is similar to a horizontally industrial case.

**Figure 8:**
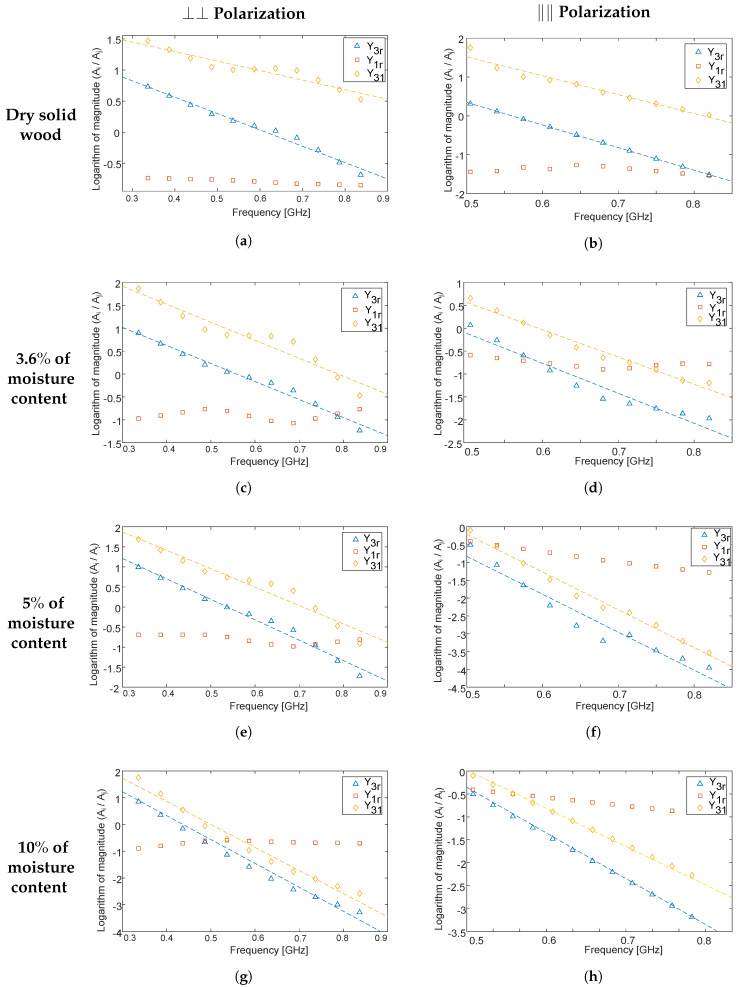
Experimental values of $Y_{3r}=ln(|\frac{A_{3}}{A_{r}}|),Y_{1r}=ln(|\frac{A_{1}}{A_{r}}|),\;\mathrm{and}\;Y_{31}=ln(|\frac{A_{3}}{A_{1}}|)$ vs. the frequency for different moisture content values (for Object A) and for polarization states ⊥⊥ and ‖‖ as shown in the figures.

In Figure 8a–h, the slopes (i.e., $Y_{3r},Y_{1r}$, and $Y_{31}$) obtained from data from Object A vs. the frequency for different moisture content values as well as for polarization states ⊥⊥ and ‖‖ are shown. To evaluate the validity of the method for determining *k* (Section 2.3), we show $Y_{3r}$, $Y_{1r}$, and $Y_{31}$.

It can be clearly seen in Figure 8 that the slope of $Y_{3r}$ and $Y_{31}$ increases with moisture content, as expected, since the attenuation of higher frequencies increases with objects’ moisture content. Further, the ratio $Y_{1r}$ has no slope, which means that it is frequency-independent, in agreement with Equation (32); the lack of slope corroborates the assumptions made in the derivations in Section 2.3. Notice that $Y_{3r}$ and $Y_{31}$ have approximately the same slope in the figures, in agreement with Equations (29) and (33), which also suggests that the assumptions made in the derivations are correct. Furthermore, the ratio with the reference pulse’s magnitude in the in denominator, i.e., $Y_{3r}=ln\left(|\frac{A_{3}}{A_{r}}|\right)$, is less noisy than $Y_{31}$ for low moisture levels, since pulse $a_{3}$ is larger than $a_{1}$. When the moisture level increases, $a_{3}$ becomes more damped compared to $a_{1}$ and $Y_{3r}$ and $Y_{31}$ have a similar noise level. However, the noise in $Y_{3r}$ depends less on the moisture level. Notice that the frequency scale of ⊥⊥ and ‖‖ polarization states are different, which is due to different noise floors in the experimental values. Finally, the investigated real and imaginary parts, i.e., $n_{⊥},k_{⊥}$ and $n_{\|},k_{\|}$ values for the different moisture content values in SW and WCs, are shown in Table 1 and Table 2, respectively.

The results of SW in Table 1 show that the values of $n_{\|}$, and $k_{\|}$ and $n_{⊥}$, and $k_{⊥}$ are not the same in the ‖ and ⊥ polarization measurements, as expected, because of the orientation of fibers in SW give rise to birefringent dielectric properties along different axes. The values for *n* and *k* in Table 1 are similar to those for several types of wood reported in [35,36]; the increase in both *n* and *k* with moisture content is also in qualitative agreement with the results in [35,36].

The results of WCs in Table 2 show that the values of $n_{\|}$ and $k_{\|}$ as well as $n_{⊥}$ and $k_{⊥}$ are approximately the same results in the ‖ and ⊥ polarization measurements, as expected; the orientation of the fiber is random in the horizontal plane due to gravity. Naturally, the refractive index for WCs, $n_{WC}$ should be in the range of $n_{air}<n_{WC}\leq n_{SW}$ and $k_{WC}\leq k_{SW}$. The values for *n* in Table 2 is smaller than those for $\epsilon^{\prime }=n^{2}$ in [10], in which the WCs were densely packed in volumes of several m${}^{3}$.

**Figure 9:**
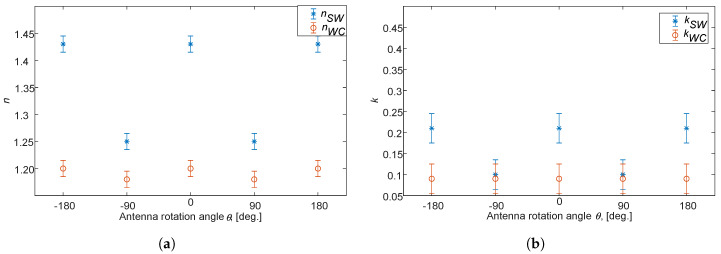
The refractive index of dry SW and dry WCs for different antenna rotation angles, (**a**) real part, *n*, and (**b**) imaginary part, *k*. Notice that *θ* = 0°, ±180° corresponds to ‖‖ and *θ* = ±90° corresponds to ⊥⊥.

Figure 9 shows a comparison of the refractive index and absorption for SW and WCs with different antenna rotation angle θ. The *n* and *k* values for SW has a clear maximum at $0^{\circ }$ (i.e., ‖‖ polarization) and a corresponding minimum at $90^{\circ }$ (i.e., ⊥⊥ polarization state of the radar. For the WCs, the variation with rotation angle is comparatively very small (and can be seen as a straight line).

### 4.2. Conveyor Belt

**Figure 10:**
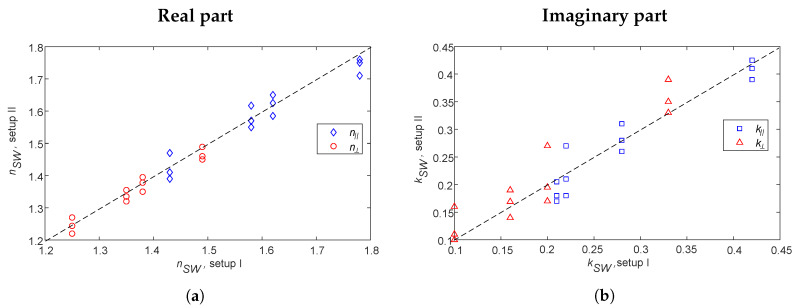

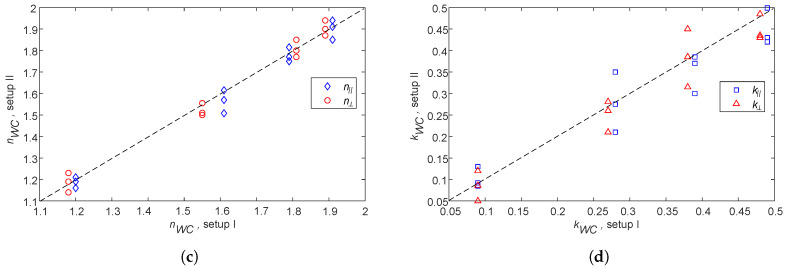
Comparison of the refractive index for SW and WCs as measured by the two different set up geometries (Setups I and II) for different moisture content values: (**a**) the real part, *n*, of SW (Object C vs. Object A), (**b**) the imaginary part, *k*, of SW (Object C vs. Object A), (**c**) the real part, *n*, of WCs (Object D vs. Object B), and (**d**) the imaginary part, *k*, of WCs (Object D vs. Object B).

Measurements were made on SW (Object C above) and WCs (Object D above) on a conveyor belt, illuminated vertically (Setup II above). In this case, $D_{3}=0$, in Figure 1 and Figure 2; the data were processed as described in Section 2.3. Notice that measurements were carried through, as described in Section 4.1. The SW and WCs were inside plastic boxes (i.e., a well-defined geometry), whereas in this case the WCs were not inside a box but directly on the conveyor belt. Hence, the upper interface was not as good of a planar surface as it was when the WCs were in plastic boxes. This case is similar to a vertically industrial case (where a large volume of samples are on the conveyor belt). In order to evaluate the data from Setup II, we compared the determined refractive index with those determined using Setup I. In Figure 10, the real and imaginary parts of SW and WCs (i.e., *n* and *k*) determined from data from Setup II are shown vs. those from Setup I. The investigated data for different moisture content are shown as well as for different polarizations. The data for the two different setups are in good agreement. Thus, we conclude that the presented method can be used also for WCs on conveyor belts when the upper interface is not shaped by a plastic lid or surface. Notice that for WCs the *n* and *k* values for the different polarizations are practically the same. The reason for this is that gravity orients the WCs such that the anisotropy is not seen when viewed vertically, as explained in [10].

## 5. Discussion

We have presented a method for determining the real and imaginary parts of the refractive index of objects from UWB radar measurements. Measuring both the real and imaginary parts enables better classification in industrial applications than measuring only the real part, which is more common. The method is insensitive to differences in object size and hardware impairments of the radar system and is therefore suitable to industrial environments that are often harsher and not as well controlled as laboratory ones.

The method enables the characterization of objects that are finite in size as long as they have flat and parallel interfaces and materials with small frequency dependence; furthermore, the frequency dependence of antennas and analog front-end does not affect the method significantly. More complicated object geometries would require more elaborate data processing and in most cases well controlled measurement conditions. Such methods include separation over overlapping pulses and methods for electromagnetic inversion of object geometries and may be of interest in future work and other applications where the objects have multiple layers or not flat parallel interfaces. The method could be further developed to incorporate frequency dependence of the refractive index. For the real part, it means that the group delay vs. the frequency of the detected pulses should be investigated. For the imaginary part, it means that not only a straight line but the variation with frequency should be determined. However, as seen in Figure 8, we could not see any significant frequency dependence in the data of the objects that we investigated.

The method could be modified or improved for specific radar systems or applications such as frequency-modulated continuous-wave radar, as long as, the reflected pulse can be separated in time domain. Clutter could be measured and subtracted in conventional ways. It was not found to be important in our case. Other types of reference objects such as trihedral reflectors could be used. The data could be processed by matching filters that compensate for the antennas’ frequency dependence. The pulses would be shorter in the time domain and, hence, more easily separable. The presented method is suitable for characterizing wood-based materials in which the dielectric properties (real and imaginary parts) change with moisture content. Other types of materials could also be used; that may require other frequency ranges, but UWB radar is now available in several frequency ranges.

## 6. Conclusions

We have presented a UWB method for determining dielectric properties (expressed as a complex refractive index) that is suitable for industrial in-line testing; the method was used on SW and WCs. A UWB radar system with a reflector as a reference and with the possibility for polarimetric measurements was used. Tests were made in which the objects were on a conveyor belt and were illuminated vertically; the objects were resting on the reference reflector. The method can be used on objects of finite size and at a finite distance; the validity of the method was validated, and a valid object size and valid distances were found. Measurements were also made with the objects illuminated horizontally and with an unknown distance to the reference reflector. The moisture content of the SW and WCs was controlled, and the complex refractive index was measured for different moisture content as well as for different polarization states (i.e., ‖‖ and ⊥⊥ polarization). The detected radar pulses from reflections in different interfaces are analyzed in the time- and frequency- domain. Furthermore, the refractive index vs. antenna rotation shows significant variation for the SW and WCs, i.e., the method enables the detection of the anisotropy of the SW and WCs, caused by the internal inhomogeneities. The results for the complex refractive index and anisotropy for SW and WCs were in agreement with similar data from the literature.

## Figures and Tables

**Table 1:** Refractive index of SW (‖ and ⊥ components) in various polarization states. The real and imaginary parts are determined for the ‖ and ⊥ polarization of the radar unit. In ‖ polarization, the EM wave is parallel to fiber orientation in wood, and so on. Radar-determined distance $D_{2}$ is 0.327±0.03 m, while the ruler-measured distance $D_{2}$ is 0.30±0.02 m.

	$n_{\|}$	$n_{⊥}$	$k_{\|}$	$k_{⊥}$
Dry SW	1.43 ± 0.03	1.25 ± 0.03	0.21 ± 0.07	0.10 ± 0.07
3.6% of moisture content	1.58 ± 0.04	1.35 ± 0.04	0.24 ± 0.08	0.16 ± 0.08
5% of moisture content	1.62 ± 0.04	1.38 ± 0.04	0.28 ± 0.08	0.20 ± 0.08
10% of moisture content	1.78 ± 0.05	1.49 ± 0.05	0.42 ± 0.09	0.33 ± 0.09

**Table 2:** Refractive index of WCs (‖ and ⊥ components) in different polarization states. The real and imaginary parts are determined for the ‖ and ⊥ polarization of the radar unit. Radar-determined distance $D_{2}$ is 0.426±0.03 m, while the ruler-measured distance $D_{2}$ is 0.40±0.02 m.

	$n_{\|}$	$n_{⊥}$	$k_{\|}$	$k_{⊥}$
Dry WCs	1.20 ± 0.03	1.18 ± 0.03	0.09 ± 0.07	0.09 ± 0.07
30% of moisture content	1.61 ± 0.04	1.55 ± 0.04	0.28 ± 0.08	0.27 ± 0.08
40% of moisture content	1.79 ± 0.04	1.81 ± 0.04	0.39 ± 0.08	0.38 ± 0.08
50% of moisture content	1.91 ± 0.05	1.89 ± 0.05	0.49 ± 0.09	0.48 ± 0.09

## Abbreviations

The following abbreviations are used in this manuscript:

EM	Electromagnetic
UWB	Ultra Wide Band
SW	Solid Wood
WCs	Wood Chips
RCS	Radar cross section
